# High-level expression of human arginase I in Pichia pastoris and its immobilization on chitosan to produce L-ornithine

**DOI:** 10.1186/s12896-015-0184-2

**Published:** 2015-07-31

**Authors:** Xue Zhang, Jin Liu, Xianhong Yu, Fei Wang, Li Yi, Zhezhe Li, Yunyun Liu, Lixin Ma

**Affiliations:** Hubei Collaborative Innovation Center for Green Transformation of Bio-resources, Hubei Key Laboratory of Industrial Biotechnology, College of Life Sciences, Hubei University, Wuhan, 430062 People’s Republic of China

**Keywords:** L-ornithine, Recombinant human arginase I, Immobilization, Chitosan, L-arginine, Transformation

## Abstract

**Background:**

L-ornithine (L-Orn), is an intermediate metabolite in the urea cycle that plays a significant role in humans. L-Orn can be obtained from the catalysis of L-arginine (L-Arg) by arginase. The *Pichia pastoris* expression system offers the possibility of generating a large amount of recombinant protein. The immobilized enzyme technology can overcome the difficulties in recovery, recycling and long-term stability that result from the use of free enzyme.

**Methods:**

The recombinant human arginase I (ARG I) was obtained using an optimized method with the Pichia pastoris GS115 as the host strain. Chitosan paticles were cross-linked with glutaraldehyde and rinsed exhaustively. Then the expressed ARG I was immobilized on the crosslinked chitosan particles, and the enzymatic properties of both the free and immobilized enzymes were evaluated. At last, the immobilized ARG I was employed to catalyze L-Arg to L-Orn.

**Results:**

The results indicated that these two states both exhibited optimal activity under the same condition of pH10 at 40 °C. However, the immobilized ARG I exhibited the remarkable thermal and long-term stability as well as broad adaptability to pH, suggesting its potential for wide application in future industry. After a careful analysis of its catalytic conditions, immobilized ARG I was employed to catalyze the conversion of L-Arg to L-Orn under optimal condition of 1 % glutaraldehyde, 1 mM Mn^2+^, 40 °C, pH10 and an L-arginine (L-Arg) concentration of 200 g/L, achieving a highly converted content of 149.g/L L-Orn.

**Conclusions:**

In this work, ARG Ι was abundantly expressed, and an efficient, facile and repeatable method was developed to synthesize high-quality L-Orn. This method not only solved the problem of obtaining a large amount of arginase, but also provided a promising alternative for the future industrial production of L-Orn.

## Background

L-ornithine (L-Orn) is a non-protein amino acid that acts as an intermediate metabolite in the urea cycle [1]. It plays a significant role in humans, and is used for the treatment of the liver diseases, to strengthen the heart function, for weight loss and to boost immunity. Based on these versatile advantages, L-Orn is widely applied in the health care and pharmaceutical industries. Thus, this field has attracted considerable attention due to the enormous market [1, 2].

To the best of our knowledge, chemical synthesis, fermentation and enzymatic synthesis are the main methods used for the preparation of L-Orn. Chemical synthesis is one of the earliest methods; its drawbacks include the complex synthesis process and high cost, which have resulted in its gradual elimination [3, 4]. Despite its low cost, the fermentation method also has several disadvantages; specifically it is difficult to separate the product from the fermentation broth due to the complex composition and because back mutations of the strains are extremely common [5]. In contrast, the enzymatic synthesis method exhibits in several areas, including its facile operation and easy purification. Moreover, the emergence of immobilized enzyme technology has contributed to its development as an economically and ecologically available biocatalyst method. Therefore, the enzymatic synthesis method is highly applicable to L-Orn preparation [6, 7].

As the raw material to synthesize L-Orn, L-arginine (L-Arg), which is able to be produced by a mature and applicable technology, can be catalyzed by arginase (EC 3.5.3.1; L-arginine amidinohydrolase). Arginase is a key enzyme in the urea cycle, and catalyses the formation of urea in the mammalian liver [8, 9]. Arginase is regarded as a prospective pharmaceutical in the near future for use in the enzymotherapy of certain types of cancers that are auxotrophic for arginine; additionally, arginase is being developed used as a bioanalytical implement to monitor arginine levels in the blood [10]. Currently, arginase is mainly extracted from animal livers and exhibits low recovery, high cost and viral contamination [11]. Bacteria such as *Bacillus caldovelox*, *Bacillus anthracis*, *Helicobacter pylori*, *Bacillus thuringiensis* and *Bacillus brevis* are another sources of arginase [12–16]. However, these sources of arginase are inclined to form inclusion bodies and difficult to purify. Therefore, we employed the more efficient and facile *Pichia pastoris* (*P. pastoris*) expression system in our work.

First, a suitable expression system was required to obtain abundant and easily purified arginase. Due to the impure product obtained using the *E. coli* expression system and the high cost and strict culture conditions of mammalian cell expression systems [17], we selected *P. pastoris* as the host for the secretory expression of the recombinant human arginase Ι (ARG Ι) in this work. The advantages of this system include the opportunity to obtain large amounts of protein and a purification process that is fairly simple and straightforward. Second, the free enzyme exhibited a high level of activity, selectivity and specificity and a lack of long-term stability, recovery and recyclability, which hampered its application [18]. This problem appears to have been solved by the advent of immobilization technology. Out of the many carriers that have been considered and studied for fixing enzymes, chitosan was of interest because it provided most of the characteristics that we needed. This natural polysaccharide possesses many superiorities, such as high affinity for proteins, biodegradability, mechanical stability and diversity of geometrical configurations; moreover it was fairly suitable for the chosen biotransformation matrix [19]. Subsequently, we investigated a method for preparation of chitosan particles and a fixation procedure for ARG Ι. The optimal conversion conditions were systematically explored, and the reusability of the immobilized enzyme was also tested under these optimum conditions.

## Results and discussion

### ARG I optimization and synthesis

The ARG Ι sequence is 1032 bp in length, and encodes a protein of 37.8 kDa. The coding region, apart from a 6 × His--tag, was modified based on the codon usage bias of *P. pastoris* and synthesized by overlapping PCR [20, 21]. Then, the synthetic fragment was cloned into the pHBM905A vector as a fusion protein with the MF-alpha leader sequence for secretory expression. The recombinant plasmid was named pHBM905A-ARG Ι.

### Expression and identification of ARG I

**Fig. 1:**
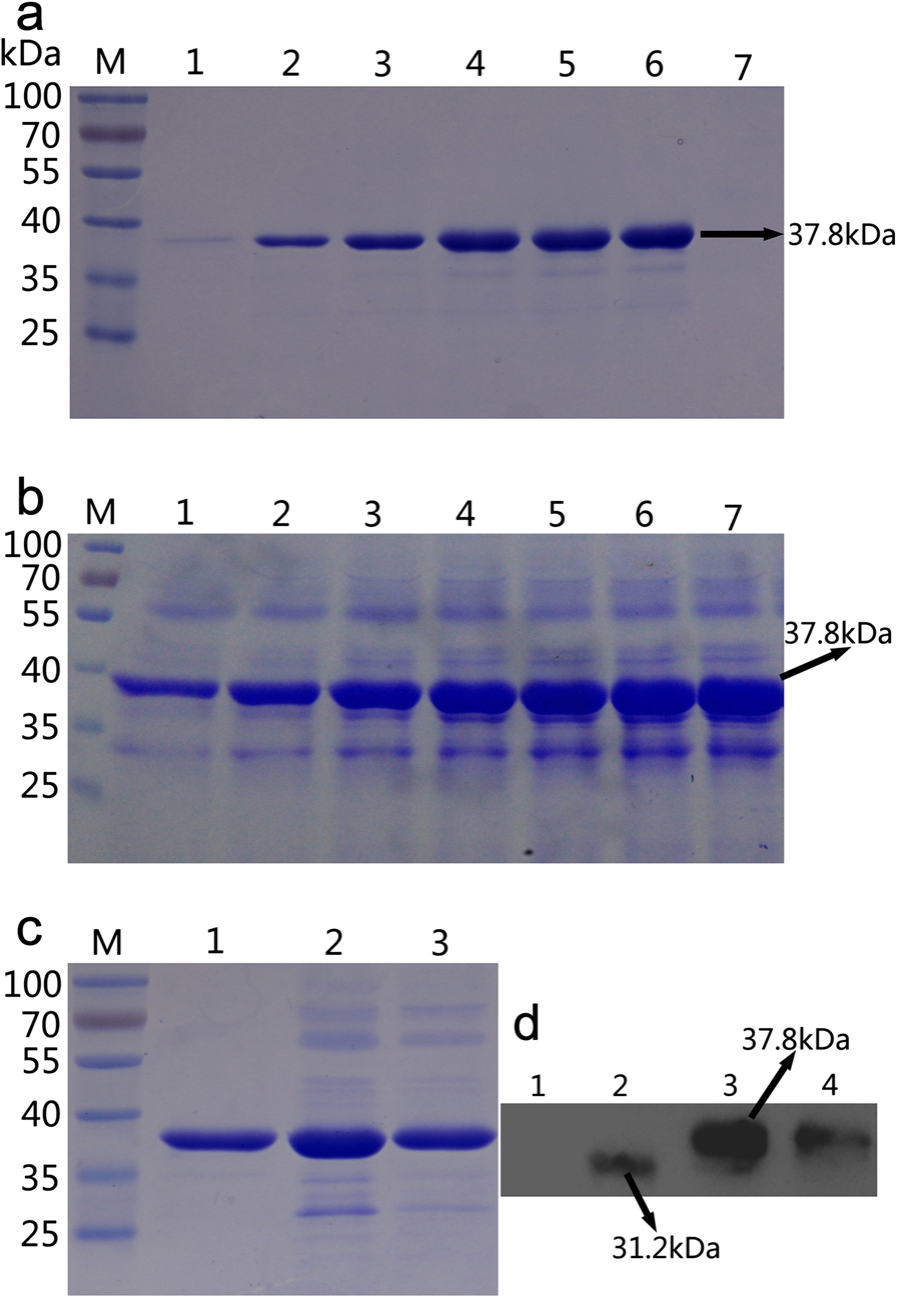
SDS-PAGE analysis of ARG I secreted in the cell culture supernatant. *M* protein molecular weight marker (the molecular weight of each band is indicated on *left*); **a** Shake-flash culture supernatants, lane 1–6, culture supernatants collected from 1 to 6 days, *lane 7*, negative control (*P. pastoris* bearing the pHBM905A vector); **b** High-density fermentation supernatants, *lane 1–7*, fermentation supernatants collected from 12 to 84 h; **c** Purified enzyme, *lane 1*, the protein purified with ultrafiltration, *lane 2*, the protein purified with Ni^2+^-affinity chromatography; **d** Western blot of ARG I, *lane 1*, negative control (*P. pastoris* bearing the pHBM905A vector), *lane 2*, positive control (Lipase from *Proteus bacillus* with a 6×His--tag), *lane 3*, the protein purified with Ni^2+^-affinity chromatography, *lane 4*, the protein purified with ultrafiltration

**Fig. 2:**
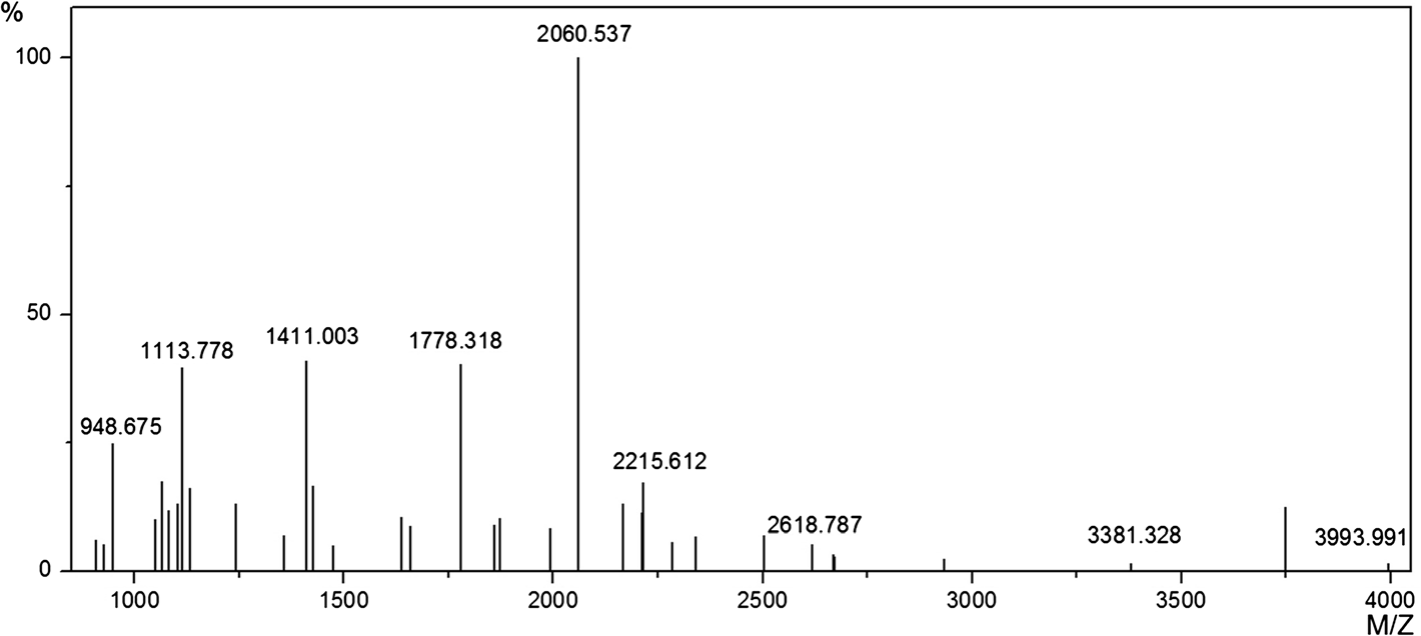
MALDI-TOF-MS peptide mass fingerprint (PMF) of ARG I generated by in-gel digestion. MALDI-TOF-MS PMF of ARG I

The recombinant plasmid pHBM905A-ARG Ι was transformed into *P. pastoris* GS115. The positive transformant, which selected from the MD plate and verified by whole-cell PCR using primers ARG-1 and ARG-40 (Table 1), was used for the expression of human arginase I. First, Western blotting with anti-6 × His antibody was used to confirm that the main band of approximately 38 kDa was Arg I (Fig. 1d). Identical amino acid sequences were obtained from this band by MALDI-TOF-MS identification (Fig. 2), confirming that the recombinant protein was human arginase I.

**Table 1:** Primers used in this study for PCR

Primers Sequence (5′ → 3′)	
arg-1 GTCAATGAGTGCTAAGTCCAGAACGATTG	
arg-2 ATGAGTGCTAAGTCCAGAACGATTG	
arg-3 CTTAGAAAATGGAGCACCAATAATACCAATCGTTCTGGACTTAGCACTC	
arg-4 GTATTATTGGTGCTCCATTTTCTAAGGGACAACCAAGAGGTGGTGTCGA	
arg-5 CCAGCCTTTCTCAAAACTGTTGGACCTTCTTCGACACCACCTCTTGGTT	
arg-6 AACAGTTTTGAGAAAGGCTGGTCTACTTGAAAAGTTGAAAGAACAAGAA	
arg-7 ATCACCGTAATCCTTAACATCACATTCTTGTTCTTTCAACTTTTCAAGT	
arg-8 GTGATGTTAAGGATTACGGTGATTTGCCATTTGCTGATATCCCAAACGA	
arg-9 GGATTCTTCACAATTTGGAATGGAGAATCGTTTGGGATATCAGCAAATG	
arg-10 CCATTCCAAATTGTGAAGAATCCAAGATCTGTGGGAAAAGCCTCTGAAC	
arg-11 CTTAACTTCAGCAACTTTACCAGCCAGCTGTTCAGAGGCTTTTCCCACA	
arg-12 GCTGGTAAAGTTGCTGAAGTTAAGAAGAACGGTAGAATTTCTCTTGTTT	
arg-13 TAGCCAAAGAATGATCACCACCCAAAACAAGAGAAATTCTACCGTTCTT	
arg-14 GGTGGTGATCATTCTTTGGCTATTGGTTCTATTTCAGGACATGCTAGAG	
arg-15 CAAATAACACCCAAGTCTGGATGAACTCTAGCATGTCCTGAAATAGAAC	
arg-16 CATCCAGACTTGGGTGTTATTTGGGTTGATGCTCATACTGACATTAACA	
arg-17 TACCAGAAGTAGTAGTCAGTGGAGTGTTAATGTCAGTATGAGCATCAAC	
arg-18 TCCACTGACTACTACTTCTGGTAACTTGCATGGTCAACCAGTTTCTTTT	
arg-19 AATCTTACCCTTCAATTCCTTCAACAAAAAAGAAACTGGTTGACCATGC	
arg-20 GTTGAAGGAATTGAAGGGTAAGATTCCAGATGTTCCAGGTTTTTCTTGG	
arg-21 ATCCTTAGCAGATATACATGGAGTAACCCAAGAAAAACCTGGAACATCT	
arg-22 TACTCCATGTATATCTGCTAAGGATATTGTTTACATCGGTTTGAGAGAT	
arg-23 AGATGTAATGTTCACCTGGATCAACATCTCTCAAACCGATGTAAACAAT	
arg-24 TGATCCAGGTGAACATTACATCTTGAAGACTTTGGGTATTAAGTACTTT	
arg-25 AACCTATCAACTTCGGTCATAGAAAAGTACTTAATACCCAAAGTCTTCA	
arg-26 TCTATGACCGAAGTTGATAGGTTGGGAATTGGCAAGGTTATGGAAGAAA	
arg-27 CTTTCTACCCAACAAGTAAGACAATGTTTCTTCCATAACCTTGCCAATT	
arg-28 ATTGTCTTACTTGTTGGGTAGAAAGAAGAGACCAATCCATTTGTCTTTT	
arg-29 AAGATGGGTCCAAACCATCAACGTCAAAAGACAAATGGATTGGTCTCTT	
arg-30 TTGATGGTTTGGACCCATCTTTCACTCCAGCTACTGGTACTCCAGTTGT	
arg-31 CAAACCCTCTCTGTACGTTAGACCACCAACAACTGGAGTACCAGTAGCT	
arg-32 TCTAACGTACAGAGAGGGTTTGTACATTACTGAGGAGATTTACAAAACA	
arg-33 ATATCCAAGCCAGACAACAAACCTGTTTTGTAAATCTCCTCAGTAATGT	
arg-34 GTTTGTTGTCTGGCTTGGATATTATGGAGGTTAATCCATCCTTGGGCAA	
arg-35 TACCGTACGAGTAACTTCTTCTGGAGTCTTGCCCAAGGATGGATTAACC	
arg-36 CAGAAGAAGTTACTCGTACGGTAAACACTGCCGTTGCAATTACATTAGC	
arg-37 GTTACCTTCACGAGCTAAACCGAAGCAAGCTAATGTAATTGCAACGGCA	
arg-38 CGGTTTAGCTCGTGAAGGTAACCATAAACCAATTGACTATTTGAACCCA	
arg-39 CACAATTTTAATGATGATGATGATGGTGCTTTGGTGGGTTCAAATAGTCAATTGGTTTA	
arg-40 GGCCATTAATGATGATGATGATGGGTGCTTTGGTGGGTTCAAATAGTCAATTGGTTTA

The results shown in Fig. 1a represent the supernatants collected from the shake-flash culture every 24 h and separated by12 % SDS-PAGE. The yield of the recombinant protein increased to reach a peak of ~121 mg/L at 144 h, after which it declined with the increasing induction time.

High-density fermentation was applied to the protein to further enhance the expression quantity. When the OD_600_ of the culture reached 300, the supply of glycerol stopped, and methanol was added to initiate the induction process. The cell wet weight increased from 180 g/L during phasing to 456 g/L at the end of methanol induction. As shown in Fig. 1b, the expression yield of ARG I reached a maximum concentration of ~1510 mg/L after 84 h of induction. Compared with the shake-flash culture, the expression quantity via high-density fermentation increased by approximately 12-fold.

Moreover, the expression quantity of recombinant human arginase I in our work was greatly improved compared with other recombinant human arginase I systems, such as *Bacillus subtilis* [22, 23], *Escherichia coli* [24, 25] and *Saccharomyces cerevisiae* [26].

### Purification of ARG I

Despite its high expression quantity, the activity of the product was restrained; therefore, the enzyme was purified using a series of methods, including ultrafiltration and Ni^2+^-affinity chromatography, to relieve the inhibition of the culture medium [27]. As shown in Table 2 and Fig. 1c, the specific activity of ARG Ι significantly increased from 8.14 to 248.4 U/mg after purification and the activity yield of the target protein was raised to 328 %. This result indicated that the elimination of inhibitors, including purines, pyrimidines, lysine, ornithine and urea, could increase the activity and we have successfully obtain the pure protein [28, 29]. Therefore, a high quantity of ARG Ι with superior purity could be synthesized using our method.

**Table 2:** Purification of ARG from 200 ml of fermentation supernatant

Step	Total protein (mg)	Total activity (U)	Specific activity (U/mg)	Yield (%)
Culture supernatant	302	2458.3	8.14	100
	78.9	8639.6	109.5	351.4
Ni^2+^-affinity chromatography	32.5	8073	248.4	328.4

### Immobilization yields

Because immobilization yields are a critical parameter in immobilization technology, a series of experiments were designed to detect them. The amount of immobilized protein was determined by subtracting the protein recovered in the supernatant from the protein subjected to immobilization. The immobilization yields were calculated as the ratio of immobilized protein to the protein subjected to immobilization. We get the results that adding 5 g (wet weight) cross-linking particles to 50 ml, 1.2 mg/ml fermentation supernatant, 47.3 % of the enzyme would be rinsed out. That is to say about 52.7 % of the enzyme was immobilized on the chitosan.

### Effect of temperature and pH on free and immobilized ARG I

**Fig. 3:**
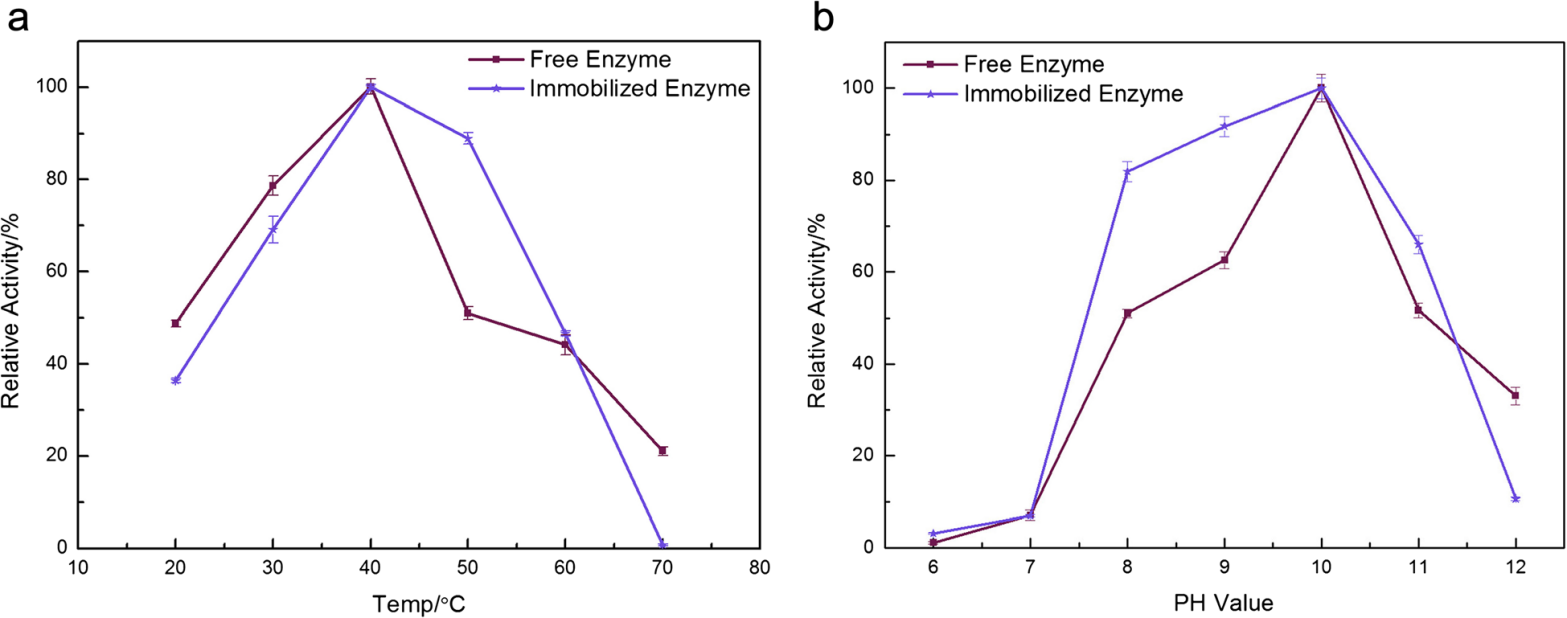
Effect of the temperature and pH on the free and immobilized ARG I. **a** Relative activity of the free and immobilized enzymes at different temperatures ranging from 20 to 70 °C; **b** Relative activity of the free and immobilized enzymes with different PH values ranging from 6 to 12; For both the free and the immobilized enzymes in (a) and (b) the maximum activity was taken as 100 %

Considering that free ARG Ι exhibits a lack of long-term stability, recovery and recyclability that limits its further application, a crosslinking method for the immobilization of ARG Ι by chitosan was employed here to improve its stability. The results of the activity of both free and immobilized ARG Ι at different temperature and pH conditions are shown in Fig. 3a and b. The activities increased and then decreased with increasing temperature and pH values; the maximum values were achieved at 40 °C and pH 10. Additionally, the relative activity of the immobilized enzyme was considerably higher than the soluble enzyme at temperatures higher than 50 °C, which suggests that immobilization of ARG Ι contributed to its temperature stability. Moreover, the soluble ARG Ι exhibited more than 60 % activity from pH 9 to 10, whereas the immobilized enzyme displayed the most activity from pH 8 to 11. This result indicates that the immobilized enzyme had a higher adaptability to pH compared to the free enzyme.

### Stability of the free and immobilized ARG I

**Fig. 4:**
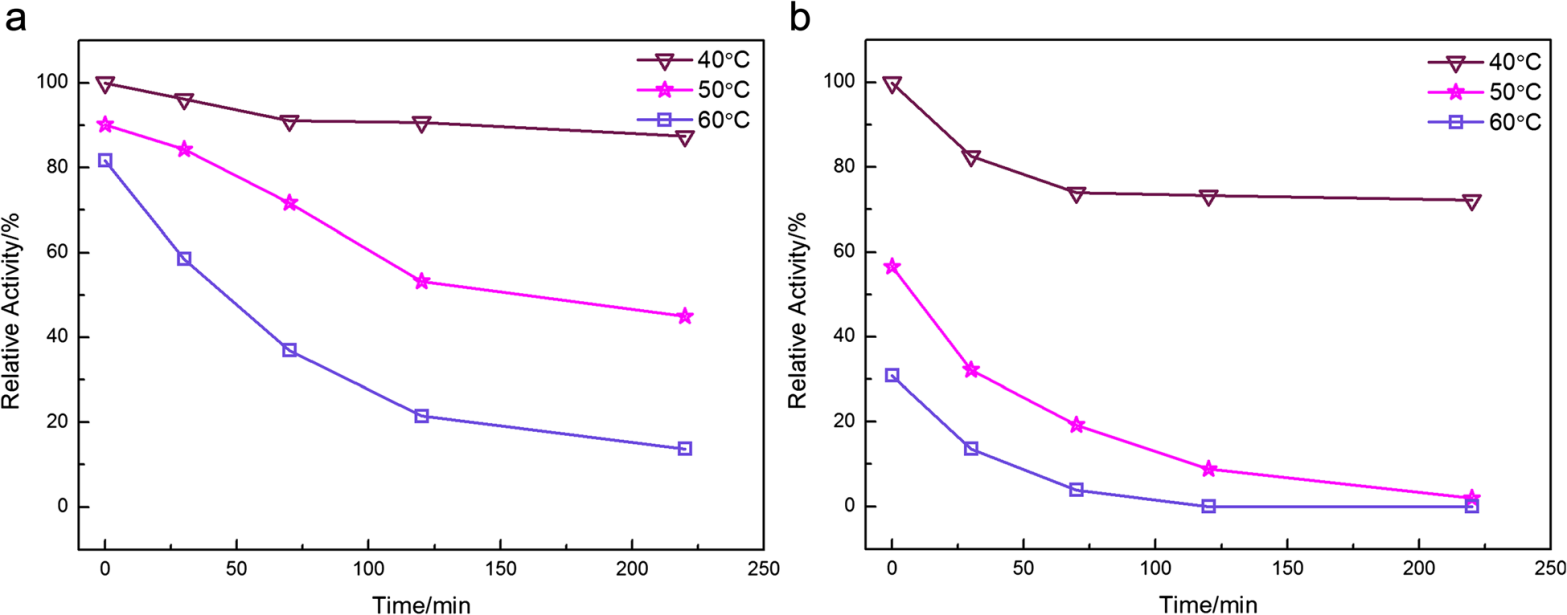
Effect of the temperature on the stability of the free and immobilized ARG I. **a** Immobilized enzyme was incubated at 40, 50 and 60 °C for 0–220 min; **b** Free enzyme was incubated under the same conditions as the immobilized enzyme; The residual activity was determined at 40 °C, pH 10. In both figure (a) and (b) the activity of the enzyme at 0 min was taken as 100 %

Both the thermal and long-term stability, which are the most important parameters for the measurement of the practicability of enzymes, of the free and immobilized ARG Ι are illustrated in Fig. 4a and b, respectively. The immobilized enzyme retained more than 85 % of its activity after incubation for 240 min at 40 °C, whereas the free enzyme lost approximately 30 % of its activity. Moreover, free ARG Ι was severely attenuated and nearly inactivated when the temperature rose from 40 to 70 °C. In contrast, the immobilized enzyme maintained more than 20 % of its activity at the high temperature. In view of these results, the immobilized ARG Ι presented remarkable enhancement in terms of thermal and long-term stability compared with the free enzyme, which is meaningful for future industrial applications.

### Optimal conditions for bioconversion

**Fig. 5:**
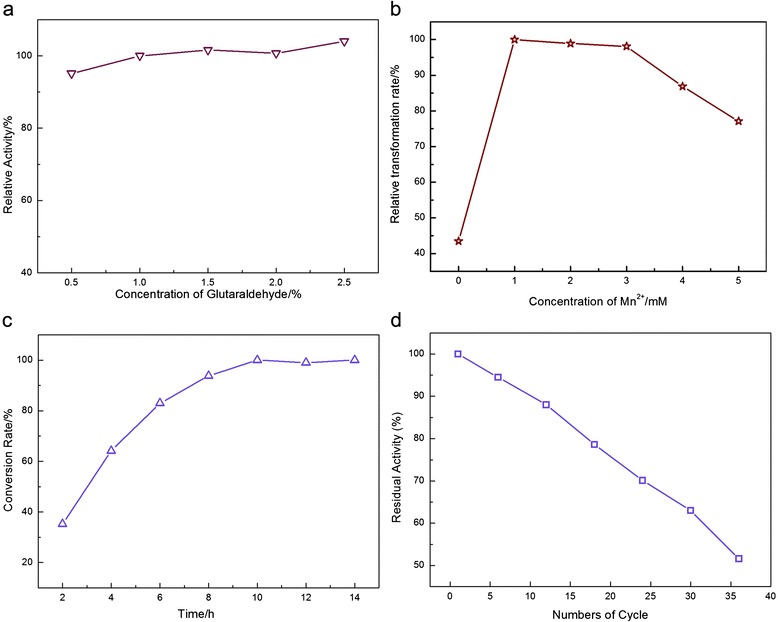
Conversion conditions of the immobilized ARG I reaction. **a** Relative activity of the arginase catalytic reaction following fixation by chitosan cross-linking with different concentrations of glutaraldehyde (ranging from 0.5 to 2.5 %), taking the activity of the concentration of 1 % as 100 %; **b** The influence of the concentration of Mn^2+^ in the reaction on the relative transformation efficiency; **c** The influence of reaction time on the conversion rate; **d** Operational stability of immobilized ARG I. All of the reactions were performed at 40 °C and pH10

Next, we carefully investigated the application of immobilized ARG Ι to catalyse L-Arg into L-Orn. Based on the series of experiments described above, the immobilized ARG Ι was utilized to synthesize L-Orn under the optimal conditions of 40 °C and pH 10. To further improve the conversion rate, the concentrations of glutaraldehyde and Mn^2+^ and the reaction time were systematically studied (Fig. 5a, b and c, respectively). According to the results from Fig. 5a, there was no significant change in the activity of the immobilized ARG Ι when the glutaraldehyde concentration increased from 0.5 to 2.5 %. This result indicates that 1 % glutaraldehyde might provide sufficient aldehyde groups on the surface of the support that are able to bind the enzyme [30]. The influence of the Mn^2+^ concentration on the conversion efficiency is presented in Fig. 5b; the best result was obtained with the use of 1 mM Mn^2+^. Finally, we determined that 200 g/L L-Arg was continually catalysed into L-Orn and reached a peak value close to 100 % after 12 h (Fig. 5c).

### Reusability of ARG I

Apart from the high thermal and long-term stability, the immobilized ARG I also possessed high operational stability in a batch reactor. The results in Fig. 5d illustrate that the activity remained at more than 50 % of its initial value after 36 cycles. Therefore, the half-life of the catalyst was determined to be 36 cycles. The results demonstrate the high operational stability of immobilized ARG Ι.

### Identification and transformation quantification of L-Orn

**Fig. 6:**
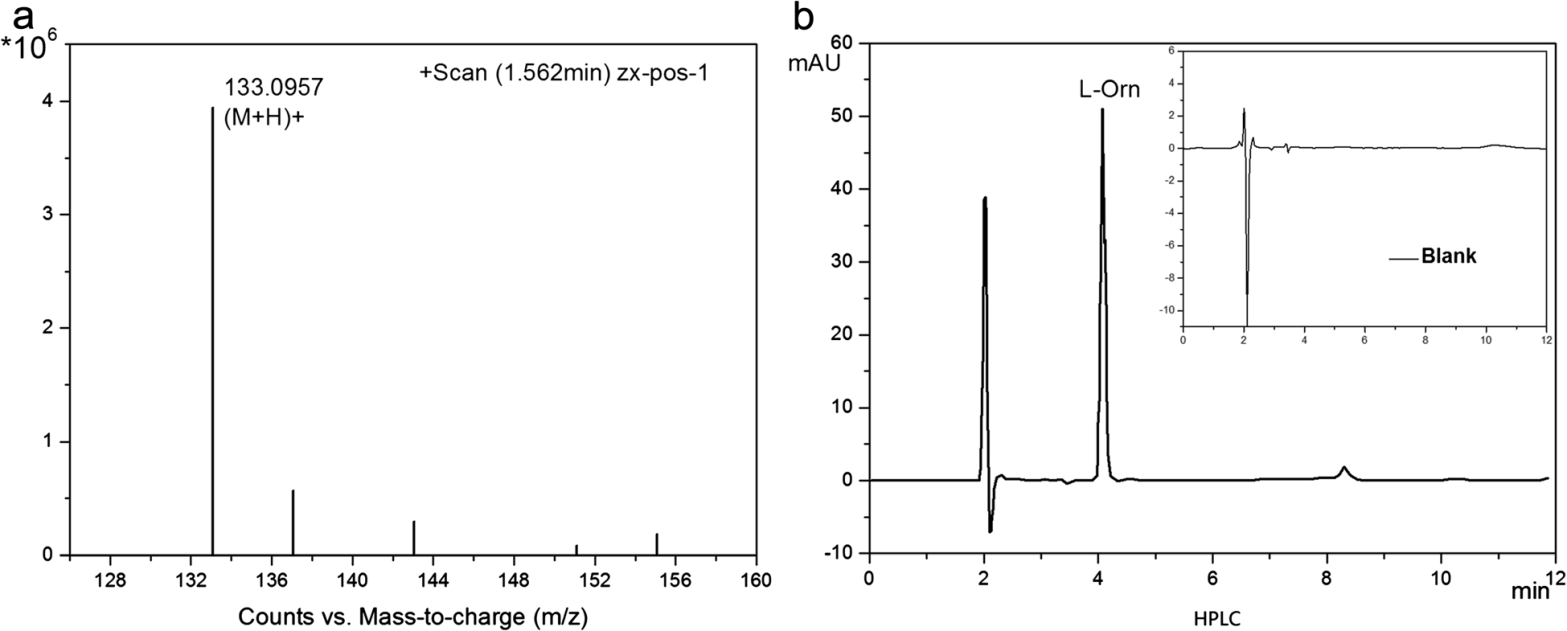
Analysis of biotransformation production. **a** Identification of L-Orn in the biotransformation by LC-MS; **b** HPLC analysis of the product of the reaction

LC-MS measurement was applied to identify the biotransformation product (Fig. 6a). The major peak at 133 is the molecular ion (M + H) + that confirms that the complex is L-Orn. Additionally, HPLC analysis was employed to quantify the L-Orn content (shown in Fig. 6b). Using the blank control in Fig. 6b as a reference, we can determine that the L-Orn peak appears at 4.072 min and that the L-Arg peak appears at 6.850 min. Based on a series of peak areas of standard samples, the L-Orn content could be calculated as 149.2 g/L and the conversion rate was 97.56 %. Thus, our method achieved a better result compared with previous reports of analogous works [31, 32].

## Conclusions

In our study, we report the development of a superior method for the expression of a high quantity of ARG I and a highly efficient immobilization method to produce high-quality L-Orn.

We employed the *Pichia pastoris* secretory expression system to express ARG I and the expression yield of it was as high as ~1510 mg/L. With ultrafiltration and Ni2 + -affinity chromatography these two steps, the specific activity of ARG Ι significantly increased from 8.14 to 248.4 U/mg and the recovery of enzyme activity raised to 328 %. Compared with other expression method, this has been a huge boost. The immobilized ARG I has been used to catalyze the conversion of L-Arg to L-Orn under an optimal condition of 1 % glutaraldehyde, 1 mM Mn2+, 40 °C, pH10 and L- Arg concentration of 200 g/L, achieving a highly converted content of 149.2 g/L L-Orn and a molar conversion of 98 %.

The results suggest that the *P. pastoris* expression system may be a suitable choice for high levels of expression of various types of proteins and can offer a more facile and efficient method to obtain pure ARG I. The technology of immobilizing enzymes on chitosan will be developed as an economically and ecologically available biocatalyst method.

## Methods

### Strains, plasmids and media

*P. pastoris* GS115, *Escherichia coli* XL10-gold and the vector pMD18-T were purchased from Invitrogen (USA). The expression vector pHBM905A is stored in our laboratory [33]. Minimal dextrose (MD), buffered minimal glycerol (BMGY) and buffered minimal methanol (BMMY) medium were prepared as described in the instructions of the P. pastoris expression manual from Invitrogen (USA).

### Reagents

Chitosan powder was purchased from Sigma (USA), and the deacetylation degree of it is about 80 %. Glutaraldehyde (25 % aqueous solution) was purchased from Sinopharm Group Co., Ltd. L-Orn and L-Arg were obtained from Biosharp (Japan). The anti-6× His monoclonal antibody was purchased from California Bioscience (USA). All other chemicals used in this study were of the highest purity commercially available.

### Optimization and synthesis of ARG I

The DNA coding sequence of arginase I from humans (GenBank Accession Np_000036) was optimized to match the codon usage preference of P. pastoris, resulting in an identical amino acid sequence with a different gene sequence. To synthesize this edited ORF, 40 oligonucleotides (Table 1) were designed with the DNA Works program (http://helixweb.nih.gov/dnaworks/) for overlapping PCR. The PCR was performed according to the method described in previous reports [20, 21]. The synthesized ARG I fragment was cloned into pMD18-T for DNA sequencing.

### Construction of the recombinant vector for expression in *P. pastoris* GS115

The full-length target gene was amplified with primers p-1 and p-40 (Table 1). Then, the amplified product was treated with T4 DNA polymerase supplemented with 1 mM dTTP to form overhangs compatible with the sticky ends of the pHBM905A vector digested with *Cpo*I and *Not*I as previously described [33]. These fragments were ligated and transformed into E. coli XL10-gold to generate recombinant vectors. The recombinant plasmid (10 μg) was linearized with *Sal*I and transformed into *P. pastoris* GS115 by electroporation (7000 V/cm, 25 μF, 400 × Bio-Rad Gene Pulser, USA) using the Life Technologies Cell Porator (USA). The positive transformants were selected on MD plates and confirmed by colony PCR.

### Expression of the ARG I and western blot assay

A recombinant *P. pastoris* GS115 bearing the target gene in its genome was incubated in 100 ml of BMGY until the OD_600_ reached approximately 20. All cells were harvested by centrifugation and transferred into 50 ml of BMMY. A total of 1 % (*v*/*v*) methanol was added every 24 h to induce the expression of the target protein. Approximately 1 ml of cell culture was collected every 24 h and centrifuged at 8000 × g for 5 min to remove cells. After 144 h, an equal volume of each supernatant was loaded onto a 12 % (*w*/*v*) polyacrylamide gel for SDS-PAGE, followed by staining with Coomassie brilliant blue G-250. The Western blot assay was implemented using the standard technique. The gels were electroblotted on polyvinylidenefluoride (PVDF) membranes using Tetra Cell (Bio-Rad, USA). For immune detection, we used a primary anti-His murine monoclonal antibody (California Bioscience, USA) and a goat anti-mouse IgG HRP-conjugated secondary antibody (California Bioscience, USA); the blots were visualized using the Bio-Rad Gel DOC^TM^ XR^+^ (USA) imaging system.

### Identification of the recombinant protein by mass spectrometry

Bands representing the target proteins on the 12 % (*v*/*v*) polyacrylamide gel were excised, and digested with trypsin overnight at 37 °C. Then identification was performed with an Ultraflex II MALDI-TOF/TOF mass spectrometer according to a previous report [34]. The mass spectrometry results were processed with the Biotools software and searched against the National Center for Biotechnology Information database through the Mascot database search engine (http://www.matrixscience.com).

### High-density fermentation

The positive colony strain was inoculated into a flask containing 200 ml of YPD medium and incubated at 28 °C and 250 rpm for 12 h; then, 200 ml of YPD medium was transferred into 2 l of BMGY medium in a − l BioFlo 2000 fermenter (New Brunswick Scientific, USA). The system was maintained at 301 K, pH 5.8, with 28 % NH_4_OH and 20 % dissolved O_2_. After the carbon source was exhausted, 12 ml of PTM1 trace salts/l (Invitrogen) with 50 % (*v*/*v*) glycerol was supplied continuously. When the OD_600_ of the cell culture reached ~300, methanol with 12 ml of PTM1 trace salts/l was added to induce the expression of ARG. The pH and temperature of the system were decreased to 5.5 and 26.3 °C, respectively. Approximately 50 ml of cell culture was collected every 12 h for SDS-PAGE.

### Purification of the ARG I

To purify the target protein, approximately 300 ml of the fermentation supernatant after induction with methanol for 84 h was centrifuged at 8000 × g for 5 min. The supernatant was collected, dialysed with a Millipore 10 kDa cut off membrane device at 4 °C to remove ions and salts and subjected to a Ni^2+^-affinity chromatography column as described in the Ni–NTA resin manual from TransGen Biotechnology (China) to remove other proteins and small peptides. The eluted solutions were dialysed with a Millipore 10 kDa cut-off membrane device.

### Enzyme activity assay

The ARG activity assay was performed using the Chinard reaction [35]. An assay reaction mixture was prepared containing 0.1 ml of arginine (0.2 M), 0.88 ml of bicarbonate buffer (50 mM, pH = 10) and 0.02 ml of a known protein concentration of purified arginase. First, each solution was incubated for 5 min at 40 °C in a water bath. Then, the mixtures were incubated at the same temperature with gentle shaking for 10 min and then moved to 100 °C for 5 min to stop the reaction. The L-ornithine content in the reaction buffer was detected using the Chinard colourimetric assay. In this study, one unit of enzyme activity was defined as the amount of enzyme that produces 1 μmol of L-ornithine/min at 40 °C. Protein contents were measured by Bradford’s method [36].

### Particle preparation

Chitosan particles were prepared by the precipitation method [37]. Chitosan (2 % *w*/*v*) was dissolved in an aqueous solution of acetic acid (2 % *w*/*v*) with magnetic stirring for 1 h. The solution was gradually added into a violently stirred coagulation liquid (2 M sodium hydroxide and 20 % *v*/*v* ethanol) until small white particles appeared. When the chitosan solution had completely changed into the white particles, they were incubated at room temperature for 3 h. Then, the obtained white granules were filtered and washed with distilled water until neutrality was reached. Cross-linking was performed by shaking the granules with glutaraldehyde solution at concentrations ranging from 0.5 to 2.5 % for 4 h; the excess glutaraldehyde was washed out with distilled water.

### Enzyme immobilization

Immobilization on chitosan microspheres was accomplished by adding 5 g (wet weight) of the cross-linked particles to 50 ml of the fermentation supernatant. The mixtures were gently stirred at room temperature for 1 h and then stored in the refrigerator overnight. Finally, the immobilized enzymes were filtered and washed with 50 mM bicarbonate buffer (pH 10) until the protein was no longer detected in the washing solution by Bradford’s method. The amount of immobilized protein [38] was determined by subtracting the protein recovered in the supernatant (P2) from the protein subjected to immobilization (P1). The immobilization yields (IY/%) were calculated as the ratio of immobilized protein (P3) to protein subjected to immobilization (P1) using the equation below:

$$ \mathrm{I}\mathrm{Y}\%=\frac{P_1-{P}_2}{P_1}\times 100\% $$

### Characteristics of the free and immobilized enzymes

Optimum temperature: the free enzyme and immobilized derivatives were incubated separately in 0.05 M bicarbonate buffer at temperatures ranging from 20 to 70 °C. Samples were collected and their activities were assessed as described above.

Optimum pH: both of the derivatives were incubated in the following buffers: phosphate buffer (50 mM, pH 6.0–7.0), Tris-HCl buffer (50 mM, pH 7.0–8.0) and bicarbonate buffer (50 mM, pH 9.0–11.0) at 40 °C. Their activities were assessed as described above.

Thermal stability: the two derivatives were incubated separately in 0.05 M bicarbonate buffer at pH 10.0 and 40, 50, and 60 °C. Periodically, samples were collected and their residual activities were assessed as described above.

### Optimum conversion conditions

Biotransformation conditions including the temperature, pH, concentrations of Mn^2+^ and substrate, amount of the immobilized enzyme, and reaction time were assessed according to the methods described in the previous study [32].

### HPLC analysis of samples and identification of the product using LC-MS

L-arginine and L-ornithine were analysed using an HPLC system (Agilent1200, USA). First, the underlying solution was removed and filtered using a 0.22 μm pore size. The solution in the reaction containing L-arginine and L-ornithine, was placed into a 1.5 ml centrifuge tube. For the chromatography, we utilized a C18 column (Luna 5 μ, 100, 150 × 4.6 mm, USA). The mobile phase consisted of 0.1 mol/L potassium dihydrogen phosphate solution: acetonitrile solution (*v*/*v* = 95:5). HPLC was performed at 40 °C using a detection wavelength of 205 nm, a flow rate of 1.0 ml/min and an injection volume of 10 μl. The product of L-ornithine was identified by LC-MS (Agilent 6224 TOF, USA).

### Operational stability of the immobilized enzyme

The operational stability of the immobilized ARG Ι was assessed using 1 g of derivative containing 82.8U/g of arginine hydrolysis. The operational conditions were pH 10.0 and 50 °C. At the end of reaction, the immobilized ARG I was removed from the reaction medium and washed with bicarbonate buffer to remove any leftovers. The activities were assessed as described above. Then, the derivative was introduced into fresh medium and subjected to another reaction [30].

### Ethics statement

This article did not involve the use of human subjects, only the gene sequence from the human liver.

## Abbreviations

L-Orn: L-ornithine
L-Arg: L-arginine
ARG I: The recombinant human arginase I
*P. pastoris*: *Pichia pastoris*
